# Genomic Instability in Human Lymphocytes from Male Users of Crack Cocaine

**DOI:** 10.3390/ijerph111010003

**Published:** 2014-09-26

**Authors:** Thiago Aley Brites de Freitas, Roberta Passos Palazzo, Fabiana Michelsen de Andrade, César Luis Reichert, Flávio Pechansky, Félix Kessler, Caroline Brunetto de Farias, Gisele Gomes de Andrade, Sandra Leistner-Segal, Sharbel Weidner Maluf

**Affiliations:** 1 Medical Genetics Service, Hospital de Clínicas de Porto Alegre, Porto Alegre, RS 90035-903, Brazil; E-Mails: thiagobrites@gmail.com (T.A.B.F.); betapalazzo@yahoo.com.br (R.P.P.); giseleandrad@gmail.com (G.G.A.); ssegal@hcpa.ufrgs.br (S.L.-S.); 2 Health Science Institute, Feevale University, RS 239, Novo Hamburgo, RS 93352-000, Brazil; E-Mails: fabiana.michelsen@hotmail.com (F.M.A.); cesar@sinos.net (C.L.R); 3 Center for Drug and Alcohol Research, Hospital de Clínicas de Porto Alegre and Universidade Federal do Rio Grande do Sul, Porto Alegre, RS 90035-903, Brazil; E-Mails: flaviopechansky@gmail.com (F.P); kessler.ez@terra.com.br (F.K.); 4 Laboratório de Pesquisas em Câncer, Hospital de Clínicas de Porto Alegre, Porto Alegre, RS 90035-903, Brazil; E-Mail: carolbfarias@gmail.com

**Keywords:** crack cocaine, drug withdrawal, DNA damage, comet assay, micronucleus

## Abstract

Recent research suggests that crack cocaine use alters systemic biochemical markers, like oxidative damage and inflammation markers, but very few studies have assessed the potential effects of crack cocaine at the cellular level. We assessed genome instability by means of the comet assay and the cytokinesis-block micronucleus technique in crack cocaine users at the time of admission to a rehabilitation clinic and at two times after the beginning of withdrawal. Thirty one active users of crack cocaine and forty control subjects were evaluated. Comparison between controls and crack cocaine users at the first analysis showed significant differences in the rates of DNA damage (*p* = 0.037). The frequency of micronuclei (MN) (*p* < 0.001) and nuclear buds (NBUDs) (*p* < 0.001) was increased, but not the frequency of nucleoplasmic bridges (NPBs) (*p* = 0.089). DNA damage decreased only after the end of treatment (*p* < 0.001). Micronuclei frequency did not decrease after treatment, and nuclear buds increased substantially. The results of this study reveal the genotoxic and mutagenic effects of crack cocaine use in human lymphocytes and pave the way for further research on cellular responses and the possible consequences of DNA damage, such as induction of irreversible neurological disease and cancer.

## 1. Introduction

Cocaine is a central nervous system stimulant extracted from the leaves of *Erythroxylum coca.* Crack is a very toxic by-product of cocaine, resulting from the addition of sodium bicarbonate to cocaine base paste, that can cause hypertension, tachycardia, muscle twitching, convulsions and even coma and death [1,2,3]. Considering the worldwide scenario of crack cocaine use, Brazil currently accounts for 20% of consumption and is the world’s largest market for crack. In 2013, consumption of crack-cocaine was 2.2% in the overall population, excluding the elderly group. Smoked cocaine use in Brazil was estimated in 1.5% for lifetime and 0.8% for last year use [4].

Crack cocaine has been reported to alter systemic biochemical markers, such as brain-derived neurotrophic factor (BDNF), IL-1β, IL-10, pro-inflammatory TNF-α and the thiobarbituric acid reactive substances (TBARS), a marker of oxidative stress that indicates the levels of lipid peroxidation [5,6]. Some studies showed that cocaine causes oxidative stress in different organs [7,8].

Very few studies have assessed the potential effects of crack cocaine at the cellular level. The potential toxic effects of cocaine and crack cocaine were studied in the protozoan, *Tetrahymena pyriformis*, and both forms of cocaine caused immunosuppression and had a significant effect on DNA content, increasing the degree of aneuploidy, with the action of crack being much more potent and rapid than that of cocaine hydrochloride [9,10,11]. Cocaine induces DNA damage in neurons from rats [12], and crack cocaine is able to induce chromosomal breakage and cellular death in oral mucosa cells of users [13].

In the present study, we assessed genome instability by means of the comet assay and the cytokinesis-block micronucleus technique (CBMN) in crack cocaine users at the time of admission to a rehabilitation clinic and in healthy, non-user controls. This initial assessment was followed by evaluation during and at the end of treatment to investigate the action of crack cocaine use on the genetic material, as well as the effect of withdrawal on the levels of DNA damage. This is the first study assessing DNA damage in peripheral blood from crack users through two techniques evaluating different types of damage and doing a follow-up evaluation during and after abstinence.

## 2. Experimental Section

### 2.1. Population

The study sample comprised 31 male active users of crack cocaine, between the ages of 18 and 45, recently admitted for treatment at the *Marcelo Campos* drug rehabilitation center, in São Leopoldo, state of Rio Grande do Sul, Brazil, and a control group of 40 participants matched by age, sex and habits from the same region. We studied only males, because this rehabilitation center accepted only male patients. The mean age was 26.12 ± 6.19 years in the test group and 29.91 ± 9.135 in the control group. Table 1 describes the sample profile. Rehabilitation was court-ordered in the majority of cases, and the expected duration of treatment ranged from 21 to 30 days.

Each participant underwent collection of 4 mL peripheral blood into heparinized tubes at three points in time: on admission, 48 h after admission and at the end of the treatment period (21 to 30 days after admission), for assessment of DNA damage by means of the comet and micronucleus assays. For the first collection, 31 subjects were allocated to the comet assay and 29 to the micronucleus assay. For the second collection, the number of subjects was 31 users, with no sampling losses, and for the third collection, the number of subjects was 23 for the comet assay and 21 for the micronucleus assay. Sampling loss occurred during the micronucleus assay at the first and third points of collection, due to unsatisfactory growth of some cultures. Furthermore, by the third point of collection, some subjects in the user group had been released from rehabilitation early and others declined collection, which reduced the sample size for both techniques. Before collection, all subjects voluntarily provided written informed consent for participation and were given a copy of the consent form, signed by the principal investigator, which contained an overview of the study. Subjects were then administered a personal health questionnaire that contained items on drug use and exposure to other potentially genotoxic agents.

All subjects were approached with the aid of a multidisciplinary team of clinic staffers, and all underwent psychiatric assessment for evaluation of cognitive functions. Subjects who were deemed unfit to provide informed consent for study participation were excluded.

### 2.2. Micronucleus Assay

Blood samples were cultured in 5 mL RPMI 1640 medium supplemented with 20% fetal bovine serum and 0.2% phytohemagglutinin per 0.5 mL sample. Culture vials were incubated at 37 °C for 44 h, followed by the addition of 4.5 g/mL cytochalasin B (Cyt B, Sigma), using the method described by Fenech and Morley in 1985 [14] and revised by Fenech and Crott in 2002 [15].

The cell suspension was fixed in a 3:1 solution of methanol in acetic acid, after standing in hypotonic KCl, and pipetted onto clean slides, which were then stained by the Giemsa method. The frequency of micronuclei (MN), nucleoplasmic bridges (NPBs) and nuclear buds (NBUDs) was scored in 2,000 binucleated lymphocytes per individual [16].

### 2.3. Comet Assay

The comet assay protocol was as described by Singh *et al.* (1988) [17]. The assay itself was carried out as described for *in vivo* samples [18,19]. Blood samples are mixed with low-melting point agarose, spread onto agarose-precoated slides, gently covered with cover slips and placed in a cold tray. Once samples have solidified, cover slips are removed and the slides left to stand in lysis buffer (2.5 M NaCl, 100 mM EDTA, 10 mM Tris, pH 10.2, to which 1% Triton X-100 and 10% DMSO are added) for 1 or 2 days, under refrigeration. Excess fluid is removed from each slide, and all slides are placed in an electrophoresis tank, to which a basic solution (300 mM NaOH, 1 mM EDTA, pH > 13) is added. Slides are left to stand in this solution for 20 min to enable unwinding of DNA and expression of alkali-labile sites and single-strand breaks. Electrophoresis is then run for 20 min at 25 V, 300 mA and 0.9 V/cm. Slides are removed from the electrophoresis tank, washed three times in neutralizing solution (0.4 M Tris, pH 7.5), rinsed three times with distilled water and left to dry at room temperature. All procedures subsequent to blood collection are carried out so as to prevent interference from light. Slides are then fixated and silver-stained as per Nadin *et al*. (2001) [20]. For assessment of DNA damage, 100 cells per sample are examined under light microscopy (×200 magnification). Cells are scored on a scale of 0 (no migration) to 4 (maximal migration) according to tail intensity (dimensions and shape). Therefore, the total sum of damage scores for a sample of 100 cells (the damage index, or D) ranges from 0 (no migration in any cell) to 400 (maximal migration in all cells). Patient and control slides are processed and analyzed together [21,22].

### 2.4. Statistical Analysis

Data were analyzed in SPSS 18.0.0, using the Generalized Estimating Equations (GEE) module for comparison between groups and experimental points. The GEE settings were distribution = binomial, link = probit and working correlation matrix = unstructured, for the micronucleus assay, and distribution = gamma, link = log and working correlation matrix = unstructured, for the comet assay. Bonferroni correction was employed. The Mann–Whitney *U*-test and Spearman rank correlation were used for comparison of simple parameters, such as age and duration of substance use. The significance level was set at 5%.

### 2.5. Ethical Considerations

All patients were submitted to standard informed consent procedures and were only allowed to sign the consent forms after a thorough review of the study objectives, limitations and patients’ rights. The study was approved by the Institutional Review Board of Hospital de Clínicas de Porto Alegre and by the National Research Council.

## 3. Results and Discussion

The characteristics of the sample are shown in Table 1.

**Table 1:** Sample profile.

	Crack Cocaine Users	Controls
N *	31	40
Age	26.13 ± 6.190	29.91 ± 9.135
Duration of crack cocaine use (months)	47.096 ± 30.48	0
Doses of crack cocaine/day **	12.98 ± 16.69	0
Last use before first blood sampling	66.7 ± 24.63	0
Cocaine use	10 (32.3%)	0 (0%)
Cannabis use	22 (71%)	0 (0%)
Tobacco use	21 (67.7%)	14 (35%)
Alcohol intake	19 (61.3%)	22 (55%)
*Ilex paraguariensis* infusion	13 (41.9%)	31 (77,5%)

* Number of individuals. All subjects (test and control) were male, as crack consumption is more prevalent amongst this gender. ** Number of crack rocks that the user is said to consume.

Comparison between controls and crack cocaine users at the first analysis showed significant differences in the rates of DNA damage (*p* = 0.037), the frequency of MN (*p* < 0.001) and nuclear buds (NBUDs) (*p* <0.001), but not in the frequency of nucleoplasmic bridges (NPBs) (*p* = 0.089) (Table 2).

**Table 2:** Frequency of micronuclei (MN), nucleoplasmic bridges (NPBs) and nuclear buds (NBUDs) and DNA damage scores (comet assay) in samples collected at admission (Time Point 1), 48 h later (Time Point 2) and at the end of treatment (Time Point 3).

	Controls	Time Point 1	Time Point 2	Time Point 3
Comet	13.05 ± 11.79 (N = 40)	29.81 ± 19.34 * *p* < 0.001 (N = 31)	22.29 ± 19.95 * *p* = 0.020 (N = 31)	10.39 ± 8.95 *** *p* = 0.004 ** *p* < 0.001 (N = 23)
MN	2.62 ± 2.26 (N = 29)	7.67 ± 4.92 * *p* < 0.001 (N = 29)	-	7.90 ± 3.32 * *p* < 0.001 (N = 21)
NPBs	0.76 ± 1.70 (N = 29)	1.51 ± 1.52 (N = 29)	-	1.95 ± 2.87 (N = 21)
Buds	1.59 ± 1.88 (N = 29)	6.27 ± 4.24 * *p* < 0.001 (N = 29)	-	16.86 ± 27.56 ** *p* = 0.009 (N = 21)

Statistically significant differences: * *versus* the control group; ** *versus* Time Point 1; *** *versus* Time Point 2. The GEE settings were distribution = binomial, link = probit and working correlation matrix = unstructured for the micronucleus assay; distribution = gamma, link = log and working correlation matrix = unstructured for the comet assay. Bonferroni correction was employed.

These findings evince the mutagenic nature of crack cocaine exposure, as an increased frequency of micronuclei is indicative of genome instability caused by established mutations beyond any possibility of repair [23]. Micronuclei are formed by chromosomal fragments produced by chromosome breakage (clastogenesis) or whole chromosome lost during cell division (aneugenesis).

**Figure 1:**
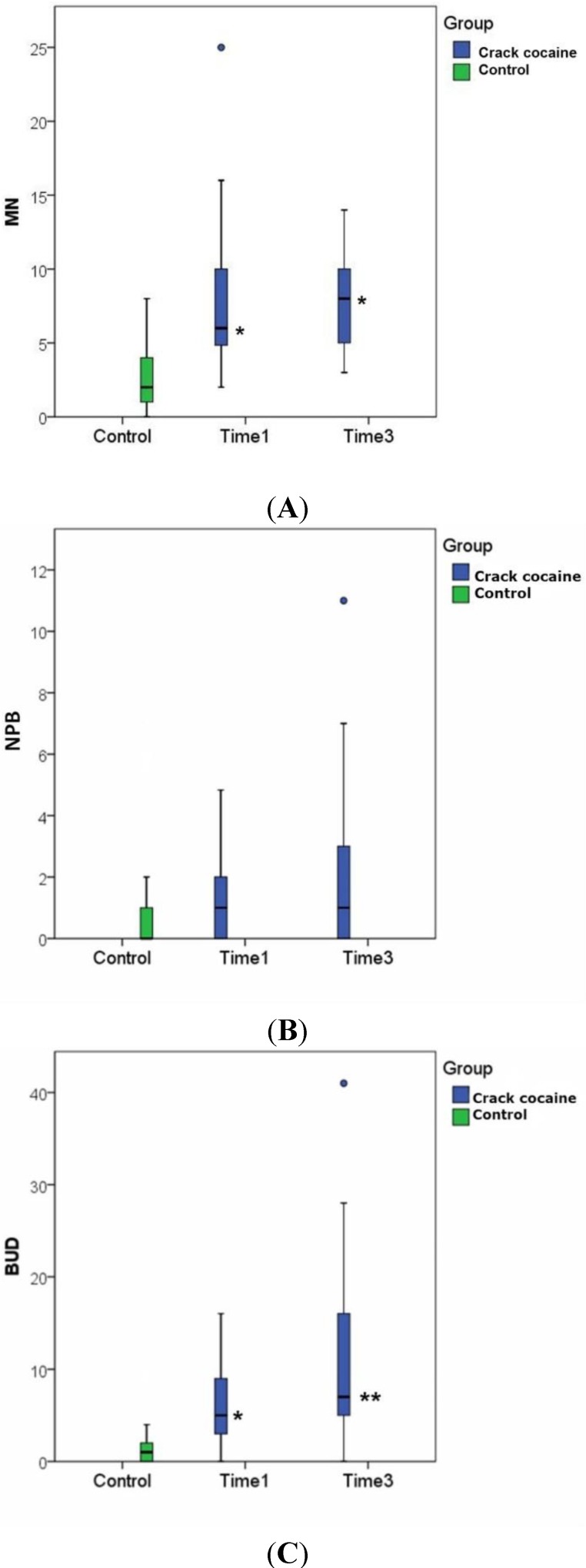
Distribution of MN (**A**), NPB (**B**) and NBUD (**C**) values at Time Points 1 and 3 and comparison with the control group; ● outliers: values that lie more than one and a half times the length of the box from the end of the box; statistically significant differences: * *versus* the control group; ** *versus* Time Point 1; Time 1: beginning of withdrawal; Time 3: at the end of the treatment period (21 to 30 days after withdrawal).

The frequency of micronuclei and nucleoplasmic bridges did not decrease after treatment, and the number of nuclear buds increased substantially (Table 2, Figure 1). These findings raise a question that lacks evidence to interpret: Was the withdrawal time not sufficient to reduce the frequency of mutagenicity markers? Future studies should be conducted to examine whether this reduction actually occurs and how long it takes for the frequencies of these markers to become similar to controls. The frequency of nucleoplasmic bridges was relatively low in view of the total number of analyzed cells. This finding may be related to the mechanism of NPB formation, which does not include aneugenesis, only clastogenesis [24]. Another key factor was the low frequency of these anomalies as compared with the MN frequency found, which is frequently observed in studies that employ these two markers [25]. Perhaps, analysis of 1000 binucleated cells is not enough for assessment of the frequency of nucleoplasmic bridges in chronic exposure studies.

**Figure 2:**
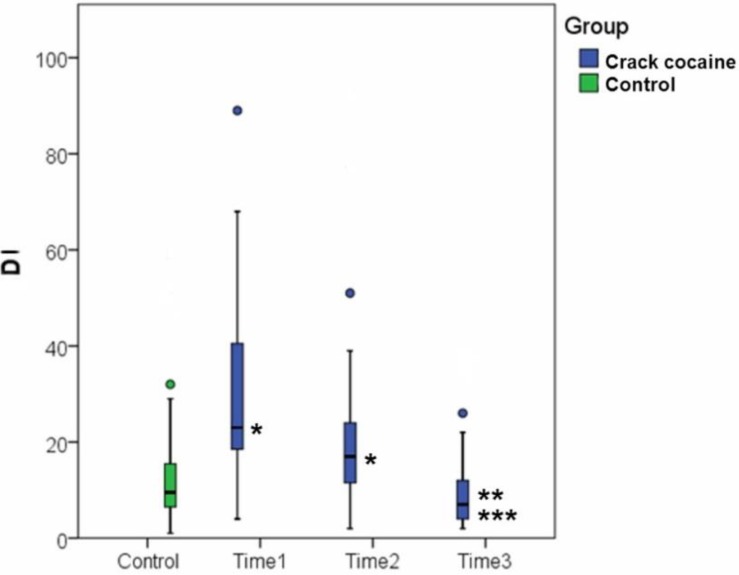
Damage index at each of the three time points of assessment and comparison with the control group; ● outliers: values that lie more than one and a half times the length of the box from the end of the box; statistically significant differences: * *versus* the control group; ** *versus* Time Point 1; *** *versus* Time Point 2. Time 1: beginning of withdrawal; Time 2: 48 h after withdrawal; Time 3: at the end of the treatment period (21 to 30 days after withdrawal).

Rates of DNA damage revealed a statistically significant decrease only after the end of treatment (*p* < 0.001) (Table 2, Figure 2). This finding revealed a reduction in reparable damage [17,26] during treatment, but 48 h was not a sufficiently long period between assessments for detection of significant differences. However, a significant difference was detected between samples collected during initial (pre-treatment) assessment and those collected at final (post-treatment) assessment. Once again, this revealed an effect of rehabilitation treatment, which reduced the rates of DNA damage as measured by the comet assay.

**Figure 3:**
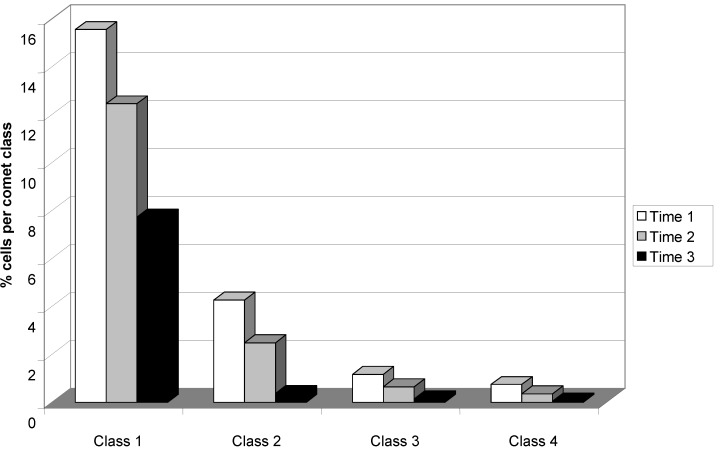
Comparison of the frequency of damaged cells of each comet class between Time Points 1, 2, and 3; Class 0: no damage; Class 1: with a tail lower than the diameter of the head (nucleus); Class 2: with a tail length from one- to two-times the diameter of the head; Class 3: with a tail greater than two-times the diameter of the head; Class 4: small head (with tail width greater than the nucleus diameter); Time 1: beginning of withdrawal; Time 2: 48 h after withdrawal; Time 3: at the end of the treatment period (21 to 30 days after withdrawal).

To evaluate the damage intensity, cells were analyzed according to the shape and size of the comet tail into five classes; Class 0: no damage; Class 1: with a tail lower than the diameter of the head (nucleus); Class 2: with a tail length from one- to two-times the diameter of the head; Class 3: with a tail greater than two-times the diameter of the head; Class 4: small head (with tail width greater than the nucleus diameter). Comparative analysis of the different comet classes, observed at the three time points, revealed a uniform decline in DNA damage indices during withdrawal of crack cocaine users. There was a reduction in all damage classes, with cells exhibiting Class 1 and Class 2 damage (minor damage) accounting for most of the DNA damage observed in this sample (Figure 3).

Correlating the medicines’ and the markers’ frequency, we cannot rule out an influence of the pharmacological treatment to which subjects were exposed at the clinic on the increase in bud frequency. This finding could be expected, as this marker is associated with gene amplification triggered by cellular toxicity [27,28]. However, some other studies show the influence of the crack withdrawal in abnormal levels of biochemical factors [5,6], which may explain this increase in bud frequency. The influence of dose, duration of use and the time of last use of crack before the first sampling (Table 1) on markers of DNA damage also showed no significant correlations. This information was obtained through a questionnaire that users answered, which can generate inaccurate data.

Overall, 71% of subjects in the test group were cannabis users. Duration of cannabis use ranged from 2 to 20 years. Non-freebase cocaine was used by 32.3% of subjects. The longest history of cocaine use was 17 years. Six subjects (19%) were, simultaneously, users of cannabis, cocaine and tobacco. Most tobacco users (81%) smoked 15–20 cigarettes per day (Table 1). Tests were conducted to assess the correlation between DNA damage markers and age, duration of crack cocaine use, time elapsed between last use of crack cocaine and first blood collection and duration of tobacco use. Furthermore, comparisons were made between subjects in the test group who drank and did not drink alcoholic beverages, those who used other illicit drugs and those who did not, those who used certain medications and those who did not and those who drank a popular local beverage (*Ilex paraguariensis* infusion) and those who did not. The following medications were used during treatment by some patients: carbamazepine (61.29%), clonazepam (benzodiazepines) (9.68%), paracetamol (acetaminophen) (3.23%), fluoxetine (16.13%), chlorpromazine (41.94%), diazepam (benzodiazepines) (16.13%), Gardenal^®^ (phenobarbital) (3.23%) lithium carbonate (9.68%). Significant findings are shown in Table 3. Note that the values of N are dependent on the number of individuals exposed to the factor analyzed, as well as certain losses occurring between sampling times.

**Table 3:** Statistically significant correlations between nuclear changes and other factors that might have an impact on DNA damage markers.

Correlation	*p*	N
*Ilex paraguariensis* infusion × MN (time point 1)	*p* = 0.045	13
Duration of tobacco use (years) × DI (time point 2)	*p* = 0.043	19
Duration of tobacco use (years) × MN (time point 1)	*p* = 0.017	17
Duration of tobacco use (years) × NPBs (time point 3)	*p* = 0.023	13
Duration of cocaine use × MN (time point 3)	*p* = 0.028	9

*p*, significance level; N, number of individuals included; MN, micronuclei frequency; DI, damage index from comet assay; NPB, nucleoplasmatic bridges. Mann–Whitney *U*-test (for *Ilex paraguariensis* consumption) and Spearman rank correlation (for the quantitative factors; the other four correlations) were used.

Regarding these other exposition factors, duration of tobacco use was significantly correlated with MN and NPB values and the damage index at different points of assessment. This finding highlights the impact of tobacco use as an enhancer factor of DNA damage, particularly when other genotoxic exposures are present, as a correlation was detected even though our sample did not include any heavy smokers; mean cigarette intake was relatively low. Toxic compounds are absorbed from cigarettes in the same manner as from crack cocaine [29]. Marcon *et al.* (2013) [30] showed that only differences in smoking habits do not contribute to a large extent to inter-individual variability in DNA damage observed in healthy human populations. However, in our study, there seems to be a synergistic effect of the tobacco and crack smoking habits.

We found the highest frequency of MN among crack cocaine users who also drank mate, a beverage made from the leaves of *Ilex paraguariensis* and widely consumed by the general population of the region. Alves *et al.* (2008) [31] found no increase in MN levels in peripheral blood lymphocytes after *in vitro* exposure of cell cultures to mate extract. However, the presence of organophosphates, such as phosphamidon, in these extracts may have exerted an additive effect that compounded the mutagenic damage associated with crack cocaine use. Phosphamidon caused considerable genetic damage in human lymphocytes from peripheral blood, *in vitro*, and in mice bone marrow, *in vivo* [32,33]. De Stefani *et al.* (2011) [34] displayed that mate drinks were positively associated with cancers of the upper aerodigestive tract, esophagus, stomach, larynx, lung, cervix uteri, bladder and kidney. However, Szymanska *et al.* (2010) [35] found a similar positive correlation only with esophagus cancer.

## 4. Conclusions

In conclusion, crack cocaine users showed significant increases in the rates of DNA damage by comet assay and in the MN frequency in the first evaluation. Rates of DNA damage quantified during the admission to the clinic revealed a decrease, which may indicate the effect of cocaine withdrawal. This effect was not observed in the MN frequency. Future studies should be conducted to examine if the withdrawal time was not sufficient to reduce MN frequency and, if this reduction actually occurs, how long it takes for the MN frequency become similar to controls.

The results of this study reveal the genotoxic and mutagenic effects of crack cocaine use in human lymphocytes and pave the way for further research on cellular responses and the possible consequences of DNA damage, such as induction of irreversible neurological disease and cancer.
